# An Unusual Case of Hemophagocytic Lymphohistiocytosis Associated with *Mycobacterium chimaera* or Large-Cell Neuroendocrine Carcinoma

**DOI:** 10.3390/curroncol30030268

**Published:** 2023-03-21

**Authors:** Tejaswi Venigalla, Sheila Kalathil, Meena Bansal, Mark Morginstin, Vinicius Jorge, Patricia Perosio

**Affiliations:** 1 Department of Internal Medicine, Albert Einstein Healthcare Network, East Norriton, PA 19141, USA; 2 Department of Hematology-Oncology, Albert Einstein Healthcare Network, Philadelphia, PA 19141, USA; 3 Department of Pathology, Albert Einstein Healthcare Network, East Norriton, PA 19141, USA

**Keywords:** hemophagocytic lymphohistiocytosis, infection, *Mycobacterium chimaera*, *M. chimera*, Neuroendocrine Cell Carcinoma, malignancy, HLH, COVID-19

## Abstract

Hemophagocytic lymphohistiocytosis (HLH) is a rare and very dangerous condition characterized by abnormal activation of the immune system, causing hemophagocytosis, inflammation, and potentially widespread organ damage. The primary (genetic) form, caused by mutations affecting lymphocyte cytotoxicity, is most commonly seen in children. Secondary HLH is commonly associated with infections, malignancies, and rheumatologic disorders. Most current information on diagnosis and treatment is based on pediatric populations. HLH is a disease that should be diagnosed and treated promptly, otherwise it is fatal. Treatment is directed at treating the triggering disorder, along with symptomatic treatment with dexamethasone and etoposide. We present a 56-year-old patient who was admitted with worsening weakness, exertional dyspnea, dry and nonproductive cough, and a 5-pound weight loss associated with loss of appetite. This is among the rare disorders that are not commonly encountered in day-to-day practice. Our differential diagnoses were broad, including infection, such as visceral leishmaniasis, atypical/tuberculous mycobacteria, histoplasmosis, Ehrlichia, Bartonella, Brucella, Adenovirus, disseminated herpes simplex virus (HSV), hematological-like Langerhans cell histiocytosis, or multicentric Castleman disease; drug reaction, such as drug rash with eosinophilia and systemic symptoms (DRESS); and metabolic disorder, including Wolman’s disease (infantile lysosomal acid lipase deficiency) or Gaucher’s disease. Based on our investigations as described in our case report, it was narrowed down to hemophagocytic lymphohistiocytosis and COVID-19. Two COVID-19 tests were negative. His lab abnormalities and diagnostic testing revealed hemophagocytic lymphohistiocytosis. He was empirically started on antibiotics and dexamethasone, to be continued for 2 weeks then tapered if the patient showed continued improvement. Dexamethasone was tapered over 8 weeks. He improved on just one of the Food and Drug Administration (FDA)-approved medications, proving that treatment should be tailored to the patient. In addition, in this case study, we included the background, etiology, pathogenesis, diagnosis, management, and prognosis of HLH.

## 1. Introduction

Hemophagocytic lymphohistiocytosis (HLH) is a very rare disease process not commonly encountered in routine clinical practice, and its presentation is even more unusual in adult patients. HLH in an adult patient is usually indicative of some insidious underlying process.

This case required both extensive workup to make the diagnosis of HLH and further evaluation to discover its etiology. Even when HLH is diagnosed and occult infection and malignancy are uncovered, it is difficult to determine which process reactively causes HLH.

A 56-year-old Caucasian man was admitted for worsening weakness and exertional dyspnea, and he was found to have HLH after an extensive workup. This disease is known to be associated with infection or malignancy, and the patient had both infection and malignancy.

## 2. Case Presentation

The patient was a 56-year-old Caucasian male who presented to the hospital with worsening weakness, exertional dyspnea, dry, nonproductive cough, loss of appetite, and a 5-pound weight loss in the 2 weeks prior. He had a significant medical history, including mitral valve repair in July 2014, status post bioprosthetic mitral valve replacement in August with culture-negative endocarditis, complicated by cerebrovascular accident, atrial flutter, tobacco abuse, and alcohol abuse.

His shortness of breath worsened quickly, with oxygen saturation dropping to 85%, and he had to be placed on bilevel positive airway pressure (BiPAP), followed by high-flow nasal cannula/noninvasive ventilation. He was then transferred to the intensive care unit (ICU) for acute hypoxemic respiratory failure. He also started to develop fevers.

## 3. Investigations

The results of pertinent investigations (Table 1) included elevated triglycerides of 383, extremely high ferritin of 9335, haptoglobin of less than 8, high lactate dehydrogenase (LDH) of 1179, and low hemoglobin of 9.4, with a hematocrit of 27.4 and low platelets of 42. He had an acute kidney injury, with uptrending creatinine from a baseline of 0.9 to 1.78. His baseline creatinine was around 0.9 to 1.0. Liver function tests (LFTs) were also elevated, with alanine aminotransferase (ALT) of 80 and aspartate aminotransferase (AST) of 234. Coagulation tests were abnormal, with elevated partial thromboplastin time (PTT) of 45.1, prothrombin (PT) of 18.2, and fibrinogen of 73. Direct Coombs was negative for hemolysis. IL-2 receptor alpha (CD25) was significantly elevated at 15,504.

On peripheral smear, the findings were as follows: red blood cell (RBC) fragments, toxic-appearing neutrophils, and decreased platelets, some of which were large. He also had moderate RBC aniso/poikilocytosis; there were no nucleated red cells or evidence of polychromatophilia. There was no evidence of rouleaux formation, but there were some teardrop-shaped cells.

He had negative blood cultures.

Acute kidney injury and microscopic hematuria prompted a vasculitis workup, including anti-neutrophil cytoplasmic antibody (ANCA), anti-glomerular basement membrane (GBM), anti-nuclear antibody (ANA), double-stranded DNA, Epstein-Barr virus (EBV), cytomegalovirus (CMV), all of which were negative.

Chest x-ray showed multifocal pneumonia.

A computerized tomography (CT) scan showed diffuse emphysematous changes, a 1.6 cm nodule in the left upper lobe of the lung, and ground-glass infiltrates.

Ultrasound of the abdomen showed hepatomegaly and splenomegaly.

Transesophageal echocardiogram (TEE) showed a large mobile echo density on the anterior mitral leaflet measuring 1.5 cm × 0.9 cm; some areas appeared calcified and were consistent with endocarditis.

**Figure 1:**
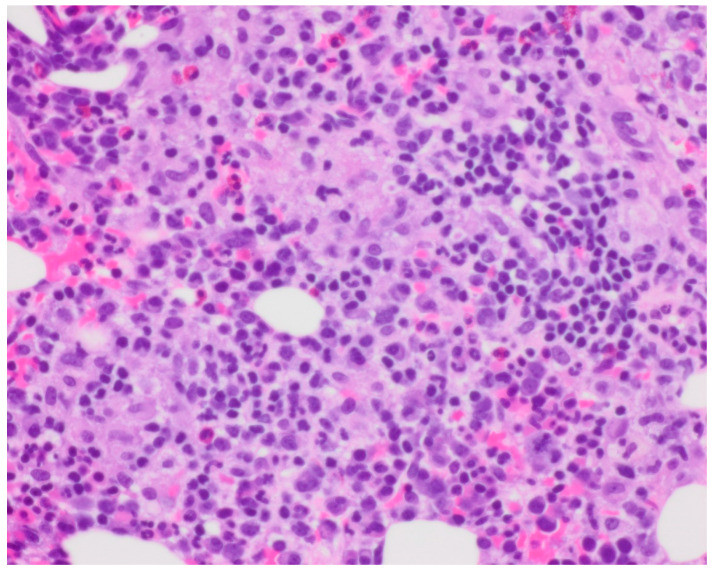
Clot section, macrophages. Hemotoxylin and Eosin (H and E) staining with 40× objective magnification.

**Figure 2:**
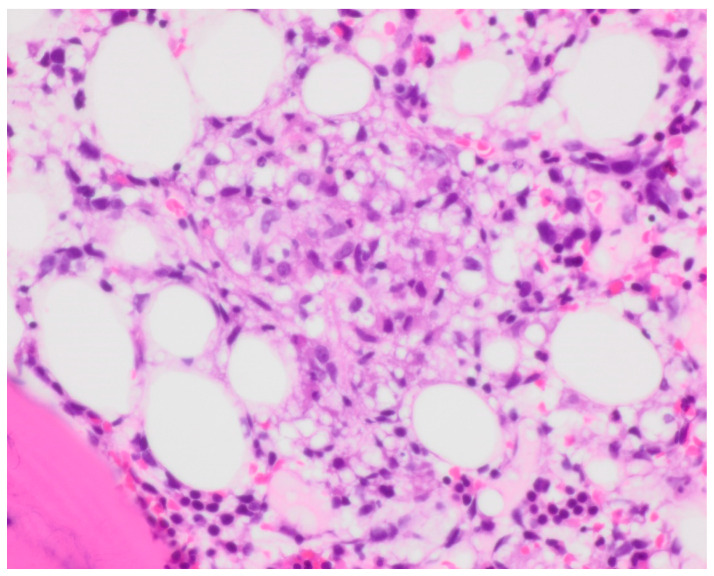
Core biopsy, showing loose aggregates of macrophages next to bone. Hemotoxylin and Eosin (H and E) staining with 40× objective magnification.

**Figure 3:**
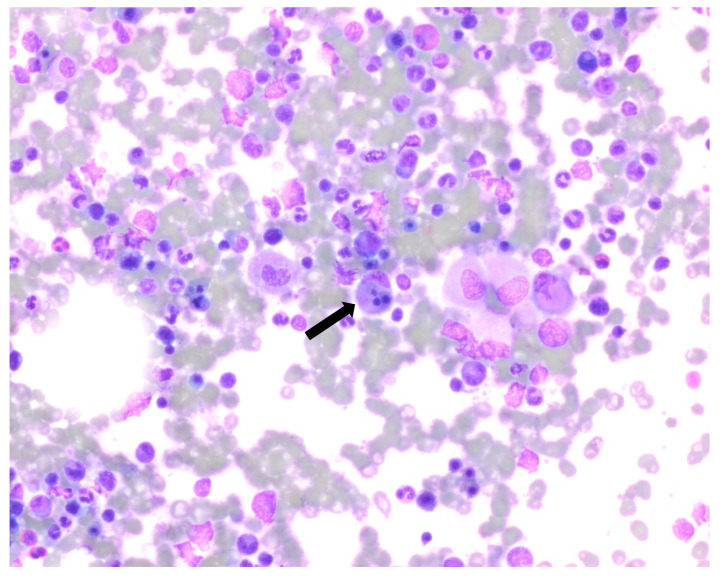
Aspirate smear, the arrow pointing marrow cells engulfed by macrophage. Giemsa stained at 100× oil immersion.

Bone marrow biopsy showed slightly hypercellular marrow with increased macrophages (Figure 1) present as diffuse infiltrates, loose aggregates (Figure 2), and tighter granulomatous aggregates. Acid-fast bacilli and fungi were negative. Occasional macrophages demonstrated the involvement of nucleated red cells and myeloid cells on the aspirate smear (Figure 3), compatible with hemophagocytosis.

Flow cytometry showed no aberrant immunophenotypic expression and no increase in blasts. NK cells represented 1% of lymphocytes. Further testing for natural killer (NK) cell function was not performed. Cytogenetic analysis showed a normal male karyotype.

## 4. Differential Diagnosis

The differential diagnoses were very broad, including infection, such as visceral leishmaniasis, atypical/tuberculous mycobacteria, histoplasmosis, Ehrlichia, Bartonella, Brucella, Adenovirus, disseminated herpes simplex virus (HSV), hematological-like Langerhans cell histiocytosis, or multicentric Castleman disease; drug reaction, such as drug rash with eosinophilia and systemic symptoms (DRESS); and metabolic disorder, including Wolman’s disease (infantile lysosomal acid lipase deficiency) or Gaucher’s disease.

For this patient, high on the differential list were hemophagocytic lymphohistiocytosis and COVID-19.

The patient had normocytic anemia, thrombocytopenia, fever, elevated serum ferritin, elevated serum triglycerides, enlarged spleen noted on ultrasound and markedly elevated soluble IL-2 receptor (CD25). According to the criteria proposed in the HLH-2004 trial, the patient met at least five of eight clinical criteria, regardless of the bone marrow changes. The presence of hemophagocytosis in the marrow is neither pathognomonic of HLH nor necessary for diagnosis; however, it supports this diagnosis in a patient who meets the other clinical criteria.

His lab abnormalities and diagnostic testing revealed hemophagocytic lymphohistiocytosis.

## 5. Treatment

Etoposide plus dexamethasone is the standard therapy for hemophagocytic syndrome.

The treatment plan for the patient was as follows:

Weeks 1 and 2: 10 mg/m^2^ daily.

Weeks 3 and 4: 5 mg/m^2^ daily.

Weeks 5 and 6: 2.5 mg/m^2^ daily.

Week 7: 1.25 mg/m^2^ daily.

Week 8: tapered to 0.

This is the schedule we followed. The treatment plan was based on the HLH-2004 protocol. The patient was empirically started on antibiotics and dexamethasone 20 mg to be continued for 2 weeks then tapered if the patient showed continued improvement.

He was not started on etoposide, as he continued to show significant improvement with dexamethasone and antibiotics. The plan was to initiate etoposide if he deteriorated or did not improve, and since he improved he never received it. He was also treated with antibiotics for suspicion of endocarditis.

## 6. Outcome and Follow-Up

The patient’s LFTs improved, his shortness of breath significantly improved, and he continued to show remarkable improvement day by day. He was discharged in stable condition.

He had a positron emission tomography (PET) scan done as an outpatient because of the lung nodule, and it showed a fluorodeoxyglucose (FDG) avid left apical nodule measuring 1.7 × 1.2 cm with a standardized uptake value (SUV) max of 7.3, suspicious for malignancy. An additional new nodule was found in the left upper lobe, which was not FDG avid and was likely infectious or inflammatory.

The patient was readmitted 2 months later for progressive weakness, fatigue, and loss of appetite. He had a Karius test done during that admission. The Karius test is a blood test that is referred to as a “liquid biopsy for infectious pathogens”. Detectable pathogens include bacteria, DNA viruses, fungi, and eukaryotes (including protozoa). The test is used to detect trace genetic material left by infecting pathogens as cell-free DNA (cfDNA). The Karius test isolates, identifies, and quantifies microbial cfDNA signals found in the blood. The testing procedure consists of (1) specimen processing, (2) next-generation sequencing, (3) sequence analysis, and (4) reporting. The patient was positive for *Mycobacterium chimaera* infection. He had a core biopsy of the lung nodule during this admission, which showed large-cell neuroendocrine carcinoma. The morphologic/cytologic features included solid nests, peripheral palisading, abundant necrosis, and large neoplastic epithelial cells; the results of histochemical tests were mucin negative, and IHC staining was synaptophysin positive (++), chromogranin positive (+), TTF1 negative (−), and P40 negative (−). These were all entirely consistent with large-cell neuroendocrine carcinoma. No other carcinoma component/type was identified in that sampling.

This suggested the possibility that the patient’s HLH could be secondary to malignancy or underlying infection (he did have findings consistent with endocarditis and a blood test positive for *M. chimaera*), or both. No genetic testing for primary HLH was performed on this patient, for unclear reasons. It is likely, as it was suspected the patient’s HLH to be secondary to either infection or malignancy, the patient presenting in adulthood, and the patient having no known family history of HLH.

This patient had multiple admissions following his initial presentation, with weakness and fatigue mostly from infectious endocarditis. However, considering his history of two sternotomies and current *M. chimaera* infection and lung malignancy, the cardiothoracic surgery team was apprehensive about the risks and benefits of a third valve replacement. He was started empirically on antitubercular medications. The radiation oncologist decided to treat with radiation therapy for the large-cell neuroendocrine carcinoma. The patient developed a pulmonary embolism during the next admission and was on a heparin drip with a transition to apixaban.

Overall, the prognosis for 6.2-year survival with HLH is 54%, but with his superimposed infection and malignancy, the prognosis was even worse. Indeed, he deteriorated further in the subsequent months. The patient and his family decided to provide him with comfort care, and the patient passed away.

## 7. Discussion

HLH has been known since as early as 1952 when it was first noted by Scottish pediatricians James Farquhar and Albert Clarieaux. It was first noticed in two infants, who passed away a few weeks after the initial presentation. They were noted to have relapsing fevers, jaundice, and purpura. Upon investigation, the physicians could not find a source of infection; the infants had complex cytopenias, splenomegaly, immature cells, and nucleated red cells on peripheral smears. The autopsy revealed hepatomegaly, splenomegaly, lymphadenopathy, and hyperactive marrow. A microscopic report from the autopsy described phagocytic histiocytes that contained one or more red cells, lymphocytes, or leukocytes. They described it as “histiocytic medullary reticulocytosis” [1].

The incidence rate of HLH is unknown, as it is quite hard to diagnose and is easily missed, and the criteria that we use are not fully established, as they were first developed for primary diagnosis in the pediatric population. A paper published in 2012 reported an incidence of 1.2 cases per 1,000,000 individuals per year [2]. A retrospective study of familial hemophagocytic lymphohistiocytosis from 1971–1986 in Sweden noted a similar incidence: 1.2 cases per 1,000,000 individuals per year [3]. A cross-sectional study in 2010 in Texas noted a prevalence of 1 in 100,000 children [4].

The median time to detection of HLH is 20 days [5].

The primary (genetic) form, caused by mutations affecting lymphocyte cytotoxicity, is most commonly seen in children [6,7,8]. Secondary HLH is commonly associated with infections, malignancies, and rheumatological disorders (Table 2).

Hemophagocytic lymphohistiocytosis (HLH) is a rare but very dangerous condition characterized by abnormal activation of the immune system, causing hemophagocytosis, inflammation, and potentially widespread organ damage [10].

The primary pathophysiology behind this condition is largely unknown; the suspected mechanism is abnormal immune activation or disruption of immunologic pathways. The cells that are thought to be causative include natural killer cells via antigen-presenting cells, and T cells. Natural killer cells use cytotoxic and cytokine-mediated mechanisms, whereas T cells use apoptosis-mediated mechanisms for immune regulation.

Genetic defects seen in HLH are related to the pathway of granule-mediated cytotoxicity. These are thought to interrupt appropriate apoptosis, causing abnormal activation of the immune system. This thus proves that interrupted apoptosis/abnormal immune cell activation is the pathophysiology behind this condition [11].

Most current information on diagnosis and treatment is based on pediatric populations. The HLH-2004 diagnostic criteria are the most commonly used; they were developed for children but are also commonly used for adults, although there is a gap in the knowledge [12]. These criteria state that a diagnosis of HLH can be established if either a molecular diagnosis is made consistent with HLH or five of eight diagnostic criteria for HLH are met. Diagnostic criteria include lab and clinical findings of fever, splenomegaly, significant cytopenia, hypertriglyceridemia and/or hypofibrinogenemia, hemophagocytosis in bone marrow/spleen or lymph nodes, low or no NK cell activity, ferritin >500 ug/L, or sCD25 > 2400 U/mL. According to these criteria, our patient met at least five of eight clinical criteria, regardless of the bone marrow changes.

Therapy based on the HLH-94 protocol consists of 8 weeks of induction therapy with etoposide (VP-16) and dexamethasone, with intrathecal therapy for those with CNS involvement. For intrathecal therapy, hydrocortisone is added to the methotrexate. Intrathecal therapy is continued until at least one week after CNS involvement has resolved, based on both clinical and CSF analysis [13].

If this induction therapy fails to improve disease activity, the treatment will be continued and the patient started on additional therapy with an allogeneic hematopoietic stem cell transplant. Treatment of the root cause is important to remove the source of insult; most commonly this involves evaluating and treating the underlying infection or malignancy.

The mortality rate associated with untreated HLH is very high. A retrospective study performed in 2014 investigated 314 adults who were suspected to have hemophagocytic lymphohistocytosis; among 162 adults who had HLH, 68 died (42%). The overall survival rate was 58%. Of the patients who did not survive, approximately half died within one month of diagnosis, especially those with hematologic malignancies. Although this study did not specify if the patients were treated for underlying disorders or with immune suppression, it suggests an overall poor prognosis for this condition [14].

Another large prospective study of 249 patients demonstrated that those treated with the HLH-94 protocol had a median survival of 54% at 6.2 years [15].

Similar case reports: A case report published in 2016 [16] is similar to our patient’s presentation, describing a 72-year-old male with a history of prosthetic aortic valve replacement (AVR) who presented with pancytopenia, fever, and weight loss. He was eventually diagnosed with HLH, with a microbiology investigation isolating *M. chimaera*.

A systematic review published in 2016 [17] evaluated the most commonly associated malignancies in HLH: Hodgkin’s lymphoma (35.2%), followed by B cell lymphoma (31.8%), with solid tumors accounting for only 3.1% of tumor types in malignancy-associated HLH. This makes the discovery of lung neuroendocrine tumors in our patients with HLH even more noteworthy.

## 8. Patient’s Perspective

The patient himself was a poor historian and apprehensive and confused about which way to proceed with his treatment. His family wanted to treat him in the best way possible considering his young age. However, his condition deteriorated over the subsequent months, and he was provided with comfort care and passed away.

## 9. Learning Points/Take-Home Messages

HLH is a disease that needs to be diagnosed and treated promptly; otherwise, it will be fatal.

Treatment is tailored based on the disease’s root cause, in addition to treatment with dexamethasone and etoposide.

Although we generally consider the most common causes of disease, it is important to have a high suspicion of the disease.

## Figures and Tables

**Table 1:** Significant Investigations.

Significant Investigations	Value	Reference Range
**Hemoglobin (g/dL)**	9.4	11.1–15.9
**Hematocrit (%)**	27.4	34.0–46.6
**Platelets (mcL)**	42	150–450
**Triglycerides (mg/dL)**	383	0–149
**Ferritin (ng/mL)**	9335	22–275
**Haptoglobin (mg/dL)**	<8	14–258
**LDH (IU/L)**	1179	125–220
**PTT (seconds)**	45.1	23.7–32.8
**PT (seconds)**	18.2	10.0–13.6
**Fibrinogen (mg/dL)**	73	200–600
**Creatinine (mg/dL)**	1.65; baseline: 0.9–1.0	0.7–1.2
**ALT (IU/L)**	80	0–55
**AST (IU/L)**	234	5–34
**IL-2 receptor alpha (CD 25) (pg/mL)**	15,504	532–1891
**Direct Coombs**	Negative	Negative
**COVID-19**	Negative x2	Negative
**ANCA, GBM, ANA, ds DNA, EBV, CMV**	Negative	Negative
**Acid-fast bacilli**	Negative	Negative

COVID-19 testing was negative twice.

**Table 2:** Common etiologies associated with HLH [9].

Infections
Viral
Epstein–Barr virus (EBV)
Cytomegalovirus (CMV)
Herpes simplex virus (HSV)
Human immunodeficiency virus (HIV)
Varicella-zoster virus
Measles
Parvovirus
Mycoplasma
Influenza A H1N1
**Bacterial**
Mycobacteria
Mycoplasma
Brucella
**Fungal:** Candida, Aspergillus
**Protozoal:** Malaria, Leishmania
**Malignancies**
Leukemia
Lymphoma (Hodgkin and non-Hodgkin)
Solid tumors (germ cell tumors)
**Rheumatological disorders**
Systemic juvenile rheumatoid arthritis (sJRA)
Systemic lupus erythematosus (SLE)
Scleroderma
Sjogren’s syndrome
Mixed connective tissue disorders
Kawasaki disease
Immune deficiency syndromes
Severe combined immunodeficiency
Common variable immunodeficiency
Chronic granulomatous disease
Stem or bone marrow transplant
Adult Still’s disease

## Data Availability

Not applicable.
